# Using the socio-ecological model in understanding antimicrobial resistance and antibiotic usage in the lakeshore communities of Calamba and Pila, Laguna, Philippines

**DOI:** 10.3389/fpubh.2026.1827837

**Published:** 2026-05-11

**Authors:** Soledad Natalia M. Dalisay, John Robert B. Magsombol, Kherson P. Gandola, Aryana Lee G. Bertuso, Aira Charlien R. De Guzman, Patrick Philip A. Salvador, Laurice Beatrice Raphaelle O. Dela Peña, Windell L. Rivera

**Affiliations:** 1 Third World Studies Center, University of the Philippines Diliman, Quezon City, Philippines; 2 Department of Anthropology, College of Social Sciences and Philosophy, University of the Philippines Diliman, Quezon City, Philippines; 3 Pathogen-Host-Environment Interactions Research Laboratory, Institute of Biology, College of Science, University of the Philippines Diliman, Quezon City, Philippines

**Keywords:** antibiotic usage, antimicrobial-resistance (AMR), lakeshore communities, socio-ecological levels, socio-ecological resilience

## Abstract

Lakes in low- to middle-income countries like the Philippines are increasingly contaminated with antimicrobial-resistant (AMR) determinants, particularly beta-lactam antimicrobials, leading to difficult-to-treat multidrug-resistant infections. Despite growing concerns, few interventions have been thoroughly evaluated to mitigate environmental contamination from hospitals, agriculture, and livestock production. To address this gap, this study employed a socio-ecological framework to examine the social science aspects of antibiotics use in selected lakeside communities in the Philippines. Specifically, it evaluated the knowledge, attitudes, and practices (KAP) related to proper antibiotic usage within lakeshore communities, identifying key challenges and opportunities for intervention. This mixed methods research employed KAP surveys, focus group discussions (FGDs), and key informant interviews (KIIs) in selected barangays of Calamba and Pila, lakeshore communities in Laguna. Data from the FGDs and KIIs were analyzed using inductive and deductive thematic analysis, while survey data were analyzed using descriptive statistics such as means and percentages. By applying the socio-ecological framework, the study contextualized how the environment, social dynamics at various core levels of the socio-ecological model, and public health policies, or lack thereof, converged to intensify AMR in these communities. The data collected, along with consultations with local government units, informed the recommendations for the development of educational tools and policies on antimicrobial use in community settings. As antibiotic misuse becomes more prevalent, this research underscores the critical need for targeted interventions to address AMR in lakeshore communities, emphasizing the importance of collaborative efforts between local government units and communities.

## 1 Introduction

A current pressing concern in public health is the inappropriate and excessive use of antimicrobial agents, which is an increasing global health problem (1). This has contributed to the emergence of antimicrobial resistance (AMR) (2), which not only increases morbidity and mortality but also has far-reaching social and economic implications (3). The consequences of AMR include prolonged illness, the need for more expensive or toxic treatments, and extended hospital stays (4).

A significant contributor to the development and spread of AMR is the consumption of animal products from livestock treated with antimicrobial substances, such as poultry, pork, and beef (5, 6, 46). In addition, environmental factors play a crucial role in the persistence and transmission of AMR. Recent studies have focused on lakes, as standing or slow-flowing bodies of water which can retain antibiotic-resistant bacteria (7). This is particularly concerning in regions where lakes serve as critical resources for agriculture and daily domestic activities. For instance, research by dela Peña et al. (8) has confirmed the presence of multidrug-resistant *Escherichia coli* in Laguna Lake, the largest lake in the Philippines, highlighting fecal and sewage sources as potential reservoirs of antibiotic-resistant bacteria. Given that lakes are essential aquatic ecosystems supporting populations in low- and middle-income countries (LMICs), the contamination of these water bodies with AMR determinants from both human and animal sources is an urgent public health issue. The increasing incidence of beta-lactam resistance has resulted in multidrug-resistant bacterial infections, posing significant challenges to healthcare systems.

Another critical factor in the spread of AMR is the lack of public awareness regarding proper antibiotic use (3). Scaioli et al. (45) found that roughly one-third (33.7%) of the population in LMICs has very limited knowledge about antibiotics and AMR. This lack of awareness has been documented in various studies, including those conducted in Bhutan (9) and India (4), where misconceptions and inappropriate antibiotic use persist.

In the Philippines, AMR remains a looming health crisis. Robredo et al. (10) assert that cultural beliefs, misconceptions, and inadequate regulation and enforcement of antibiotic use have influenced the attitudes and behaviors of both healthcare providers and patients. Self-medication is widespread, with 31–66% of Filipinos relying on it (11), as it provides an accessible—albeit risky—form of healthcare amid poverty and limited access to medical consultations and diagnostics. Moreover, many Filipinos continue to seek treatment from traditional healers (11), some of whom may administer herbal treatments containing suboptimal levels of antibiotics, further contributing to the development of AMR.

Given these concerns, public health education and awareness campaigns on AMR need to be significantly strengthened. However, for these initiatives to be culturally sensitive, effective, and appropriate, baseline evidence on antibiotic use in communities must first be gathered. Using the Socio-Ecological Model (SEM) as a guiding framework, AMR can be understood as a complex and multidimensional public health problem shaped by interacting factors across individual, interpersonal, community, institutional, and policy levels. At the individual level, AMR is influenced by inappropriate antibiotic use, including self-medication, incorrect dosage, and incomplete treatment, often driven by limited knowledge and misconceptions about antibiotics and resistance. These behaviors are further shaped at the interpersonal level through the influence of family members, peers, and healthcare providers, as well as traditional healers who may contribute to antibiotic use practices outside formal medical guidance. At the community level, cultural beliefs surrounding illness and healing, widespread access to antibiotics without prescription, and environmental contamination such as the presence of multidrug-resistant bacteria in water bodies and the contribution of livestock production systems to antimicrobial exposure collectively facilitate the persistence and spread of resistance. Institutional factors further intensify the problem, particularly through weak enforcement of antibiotic regulations, inappropriate prescribing practices, insufficient diagnostic capacity, and the routine use of antimicrobials in animal agriculture. At the policy level, gaps in regulation, surveillance, and antimicrobial stewardship programs as well as challenges in implementing national action plans such as the Philippine National Action Plan (PNAP) on AMR reflect broader structural constraints in addressing the issue. Taken together, these interconnected layers demonstrate that AMR is not solely a clinical concern but a socio-environmental phenomenon requiring coordinated interventions across all levels of the SEM to effectively mitigate its growing public health impact.

This study therefore examined the socio-environmental factors influencing antibiotic misuse and overuse in communities in the Philippines, contributing to the growing body of research on AMR awareness and mitigation strategies. Specifically, this research sought to: (1) investigate the existing knowledge, attitudes, practices, as well as culturally defined meanings relating to antimicrobial use in urban and rural communities in the Philippines; (2) provide strategies for socially and culturally aligned interventions; and (3) develop recommendations to the PNAP on AMR.

## 2 Methodology

### 2.1 Research design and methods

This mixed methods study explored the knowledge, attitudes, and practices of lakeshore communities regarding antibiotic use in selected barangays of Pila and Calamba City, Laguna. The research design incorporated a Knowledge, Attitudes, and Practices (KAP) survey, Focus Group Discussions (FGDs), and Key Informant Interviews (KIIs). The SEM guided the development of the questionnaires and interview guides.

The KAP survey utilized purposive sampling of 120 male and female participants. Participants were recruited based on the following inclusion criteria: (1) adult residents (18 years old and above) of the selected barangays in Pila and Calamba, Laguna; and (2) individuals who had taken antibiotics in the past or were currently using antibiotics. These criteria were used to ensure that participants had relevant experiences and knowledge regarding antibiotic use, which is central to the objectives of the study. The exclusion criteria included: (1) individuals who, due to physiological or communication-related limitations, were unable to respond to the survey questions; (2) transients and, (3) those who cannot recall antibiotic usage. These exclusions were necessary to ensure ethical participation, reliability of responses, and relevance to the study objectives.

The questionnaire included socio-demographic information, and sections on knowledge, attitudes, and practices related to antibiotic use. Items were adapted from previously published KAP instruments on antibiotic use and antimicrobial resistance and were refined based on relevance to the local context. Response categories for knowledge items were primarily Yes/No/Undecided; however, we acknowledge that some items (e.g., “Can antibiotics cure viral infections?”) may reflect both knowledge and beliefs, which is a limitation considered in interpreting the findings. Content validation of the questionnaire was done among researchers for relevance, clarity and completeness. Pretesting was done in a separate site among a small sample to determine understandability and logical flow. Pretesting revealed that participants were more familiar with the term “antibiotics” than with the broader category of “antimicrobials,” informing adjustments to wording. Minor revisions were also made to ensure the use of prevailing local health terminology.

FGDs were conducted in two barangays in each city/municipality with participants of varying age and sex. Discussions focused on practices related to antibiotic use, the personal and shared meanings behind these practices, and the social networks that supported them. KIIs were conducted with municipal or city Health Officers, barangay health workers from rural health units, and pharmacists, focusing on antibiotic use within their communities. The use of multiple methods permitted triangulation and cross-validation of findings.

Data were analyzed using thematic analysis. KAP survey data were analyzed using descriptive statistics, to summarize participants’ socio-demographic characteristics and antibiotic-related KAP. These analyses provided an overview of the communities’ perspectives on antibiotic use and AMR. Data from FGDs and KIIs were subjected to inductive and deductive thematic analysis. Through inductive and deductive coding, themes and subthemes related to antibiotic use and AMR were identified, systematically organized, and examined to explore relationships and uncover key findings.

The study was conducted from September 2023 to October 2024, encompassing 3 months dedicated to data collection and fieldwork. The study protocol was reviewed and approved by the College of Social Sciences and Philosophy Ethics Review Board, University of the Philippines Diliman, with the code, CSSPERB-2023-082.

### 2.2 Conceptual framework

The SEM guided the analysis of data [CDC, 2017 as cited in (12)]. The nested SEM is useful for understanding how multiple levels of social and environmental influence shape individual health behaviors and outcomes. It has been applied to examine behavioral drivers in different public health contexts, including tobacco use among airmen (13), risky sexual behavior among young adults (14) and routine childhood immunization (15). The SEM is also a useful tool in the design of comprehensive, multifactorial intervention strategies such as the toolkit for addressing obesity disparities by the Centers for Disease Control and Prevention (CDC) in the United States (16). In AMR research, the SEM provides a comprehensive perspective in identifying areas for resilience building (17). Léger et al. (18) showed how the SEM coupled with the One Health perspective can be a powerful lens in investigating the diversity of potential AMR interventions at various levels within the SEM. Moreover, SEM offers a perspective that extends beyond individual behavior. It allows the examination of the social, economic, political, and cultural contexts that shape and influence health behaviors.

The SEM emphasizes the embeddedness of individuals within different levels of systems in the social environment, the interplay among these different levels that shape human behavior surrounding health, and how these dynamics ultimately influence health outcomes. The intrapersonal or individual level refers to personal characteristics such as socioeconomic status, knowledge, attitudes, and behaviors that influence an individual’s health behavior. Interpersonal level drivers cover social networks and other relationships that affect health behaviors. Organizational drivers include workplace policies or practices and how these impact AMR-related practices. The community level focuses on the environment in which individuals live, work, and interact as well as other members in the wider community of an individual. Finally, the policy level covers formal policies and regulations that facilitate rational use of antimicrobials as well as socioeconomic conditions that create or exacerbate health inequities contributing to AMR.

**Figure 1:**
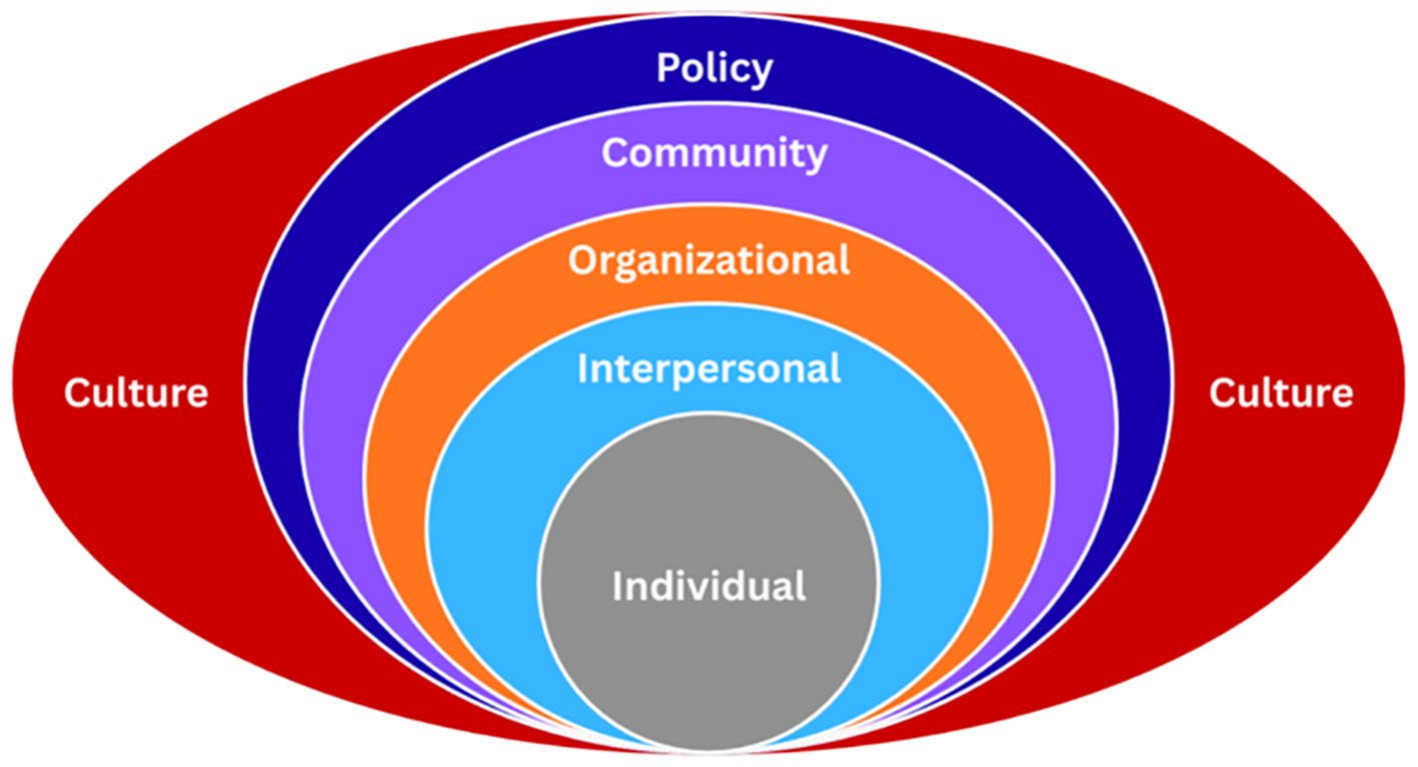
The socio-ecological model (12).

Figure 1 shows the conceptual diagram of the SEM as adopted in this study. There are many iterations of the SEM; however, the CDC model as adapted by Jordan (12) was used in this study because it incorporates the broader cultural context that forms the basis for all factors within the different socio-ecological levels. Culture provides the rationale and defines the norms of behavior within the SEM.

### 2.3 Study sites

**Figure 2:**
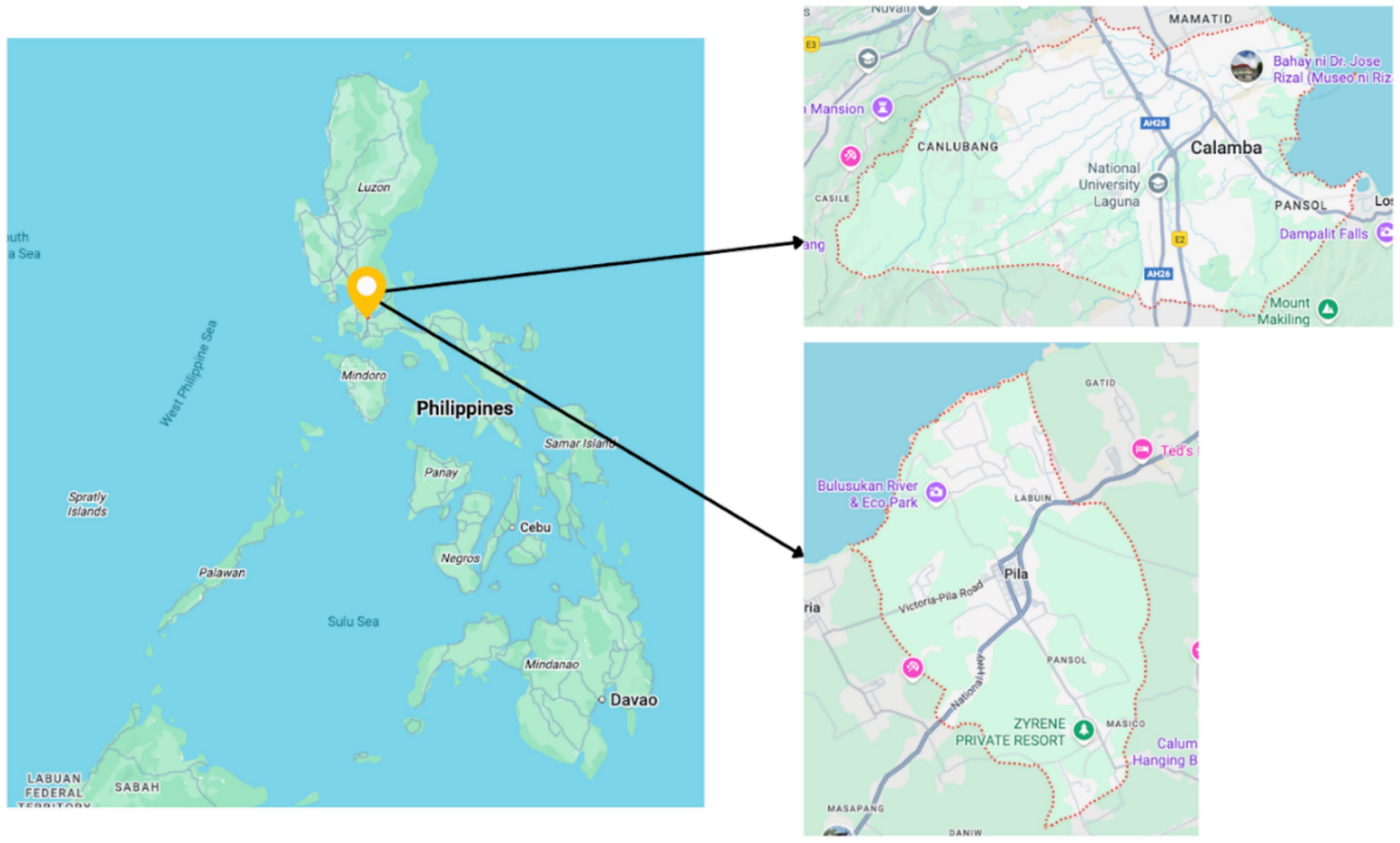
Map of the Philippines showing the study sites.

To facilitate the implementation of this study, two research sites in the province of Laguna were selected: Pila and Calamba City (Figure 2). These locations represented distinct socio-environmental contexts, with Pila serving as the rural lakeshore community and Calamba as the urban counterpart (19). The selection of these sites was based on their proximity to the lake and their potential to support a comparative analysis.

Pila is a third-class municipality in the Province of Laguna with a predominantly agricultural landscape and a well-known poultry farming industry. It has a land area of 31.20 square kilometers, accounting for about 1.62% of the province’s total area, and is strategically located within the Pasig–Laguna Lake Basin at a low elevation of approximately 7.1 meters above sea level. As of the 2020 Census, it has a population of 54,613, representing 1.61% of Laguna’s population. It has a relatively high population density of about 1,750 persons per square kilometer, reflecting its semi-urban character and proximity to major growth centers in CALABARZON and Metro Manila. CALABARZON refers to the provinces of CAvite, LAguna, BAtangas, Rizal and QueZON. Pila is administratively divided into 17 barangays, with Pinagbayanan, Santa Clara Sur, Labuin, and Bagong Pook among the most populous, indicating uneven but concentrated settlement patterns. Demographically, Pila has a young population, with a median age of 25 and a large working-age group (63.96%), suggesting a substantial labor force alongside a moderate dependency burden. Household size has gradually declined over time, averaging 4.39 members per household in 2015, consistent with broader demographic transitions. Socio-economically, Pila demonstrates steadily improving fiscal capacity, with annual regular income rising to over ₱104 million in 2016, reflecting growth in local revenue generation and national transfers (44). Its economy and settlement patterns are influenced by its accessibility to nearby cities such as Calamba and San Pablo, as well as its proximity to Laguna Lake and surrounding upland and lacustrine landscapes, which shape local livelihoods, mobility, and development trajectories.

Calamba is a first-class city in Laguna characterized by a high degree of urbanization and extensive commercial and recreational infrastructure. The city covers a land area of 149.50 square kilometers, accounting for 7.75% of the province’s total area, and lies at a relatively low elevation averaging about 15.8 meters above sea level within the Pasig–Laguna Lake basin. As of the 2020 Census, the city had a population of 539,671, representing 15.96% of Laguna’s total population and 3.33% of the regional population, with a high population density of approximately 3,610 persons per square kilometer. The population is distributed across 54 barangays, with Canlubang, Mayapa, and Looc among the most populous, reflecting uneven urban growth driven by industrial and residential expansion. Demographically, Calamba has a young population structure, with a median age of about 26 years and 68.3% of residents belonging to the economically active age group, resulting in a relatively moderate dependency ratio of 46 dependents per 100 working-age individuals. Household size has steadily declined over time, averaging 3.66 members per household in 2015, indicative of urbanization and changing family structures. Socioeconomically, Calamba is a major growth center in Laguna, evidenced by its steadily increasing annual regular income, which reached approximately ₱2.69 billion in 2016, driven by a combination of locally sourced revenues and national government shares (43). Its strategic location near Laguna Lake, Mount Makiling, and major urban centers, as well as its proximity to Metro Manila at about 46.5 kilometers, further underpins its role as a densely populated, economically dynamic, and environmentally significant urban-industrial city. Notably, there were more pharmacies in Calamba compared with Pila.

## 3 Results

### 3.1 Socio-demographic and economic profiles of participants

#### 3.1.1 KAP survey

A KAP survey was conducted among 131 respondents to examine antibiotic use and AMR awareness in two lakeshore communities in Laguna: the rural municipality of Pila (*n* = 65) and the urbanized city of Calamba (*n* = 66). In Pila, 30 males and 35 females from the 2 barangays participated, with ages ranging from 18 to 64 years (mean age of 44). In Calamba, 35 males and 31 females from two barangays were surveyed, with ages also ranging from 18 to 64 years (mean age of 46). By assessing these population groups, the study aimed to identify variations in knowledge, perceptions, and behaviors related to antibiotic use across different age groups and urbanization levels, providing essential data to address antibiotic misuse and its contribution to the emergence of AMR.

#### 3.1.2 Focus group discussion

FGDs were conducted in Pila (*n* = 25) and Calamba (*n* = 27). In Pila, the discussions involved four males (mean age = 44) and 21 females (mean age = 49). In Calamba, 10 males (mean age = 34) and 17 females (mean age = 43) participated. Overall, the ages of participants ranged from 18 to 72 years, with overall mean ages of 48 in Pila and 40 in Calamba.

#### 3.1.3 Key informant interviews

Four pharmacy assistants served as key informants across the study sites. None had any formal training in pharmacy. In Pila, two of the assistants were high school graduates, and one had completed a vocational course in garments technology. In Calamba, the assistant had completed units in Psychology and held a Pharmacy Services NC III certificate from the Technical Education and Skills Development Authority (TESDA). Four Barangay Health Workers (BHW), two from each study site, were also interviewed. Only one health professional in Pila agreed to participate in the interview.

### 3.2 Socio-ecological drivers of AMR

#### 3.2.1 Intrapersonal level

- *Knowledge, Attitudes, and Practices on Antibiotic Use*

**Figure 3:**
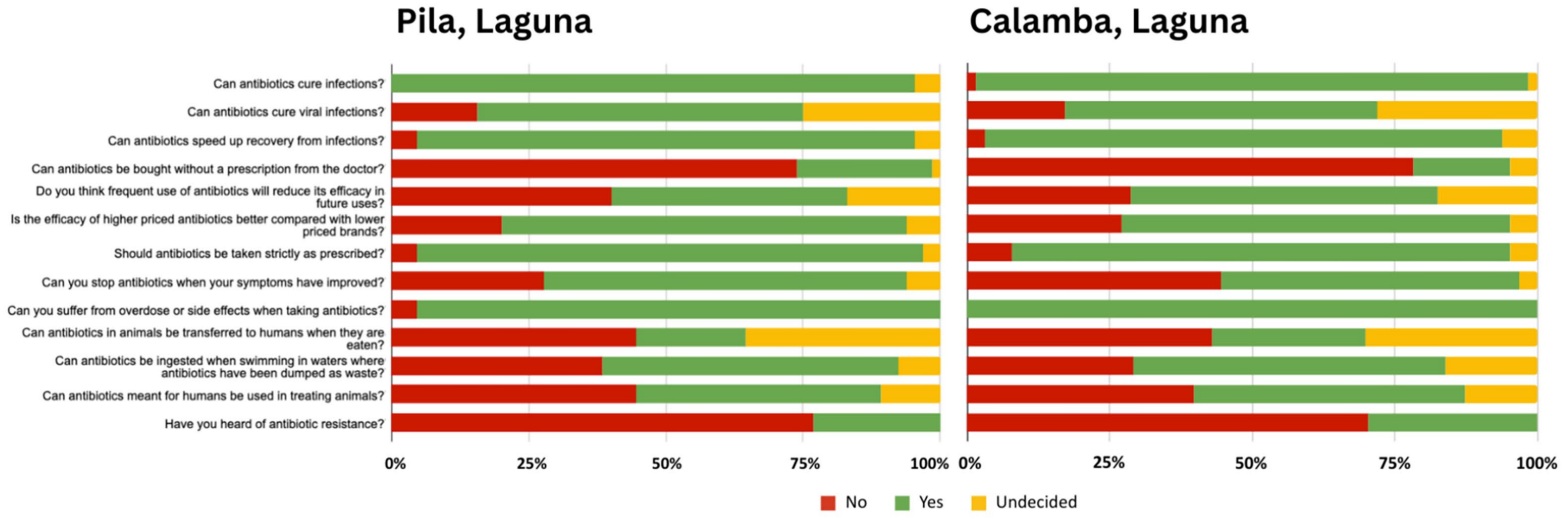
Comparative knowledge on antibiotic use and resistance in Pila and Calamba, Laguna.

**Figure 4:**
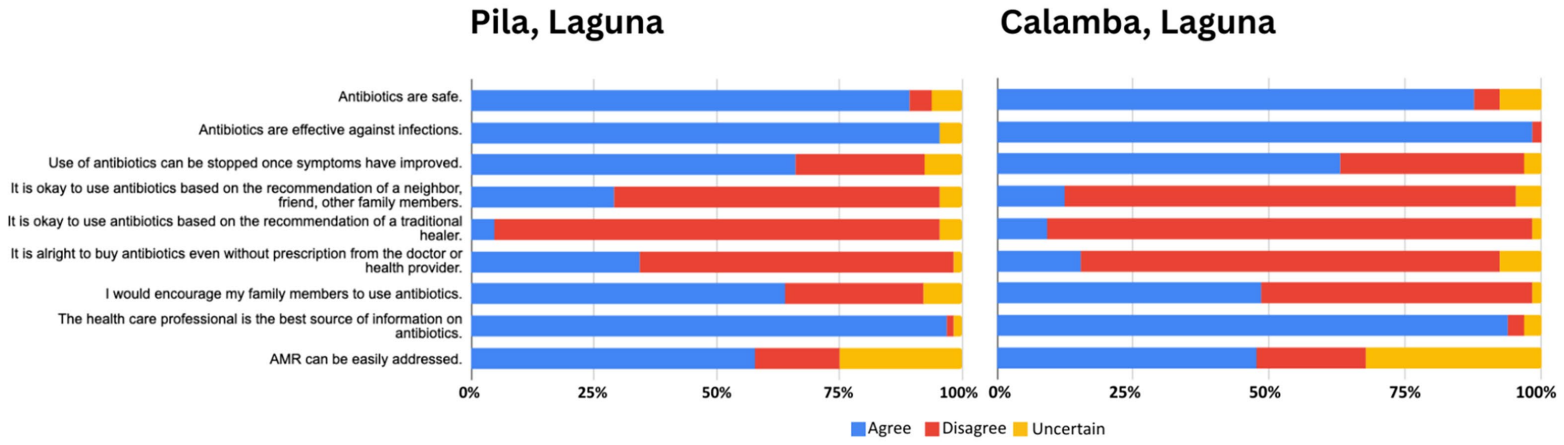
Comparative attitudes toward antibiotic use and resistance in Pila and Calamba, Laguna.

**Figure 5:**
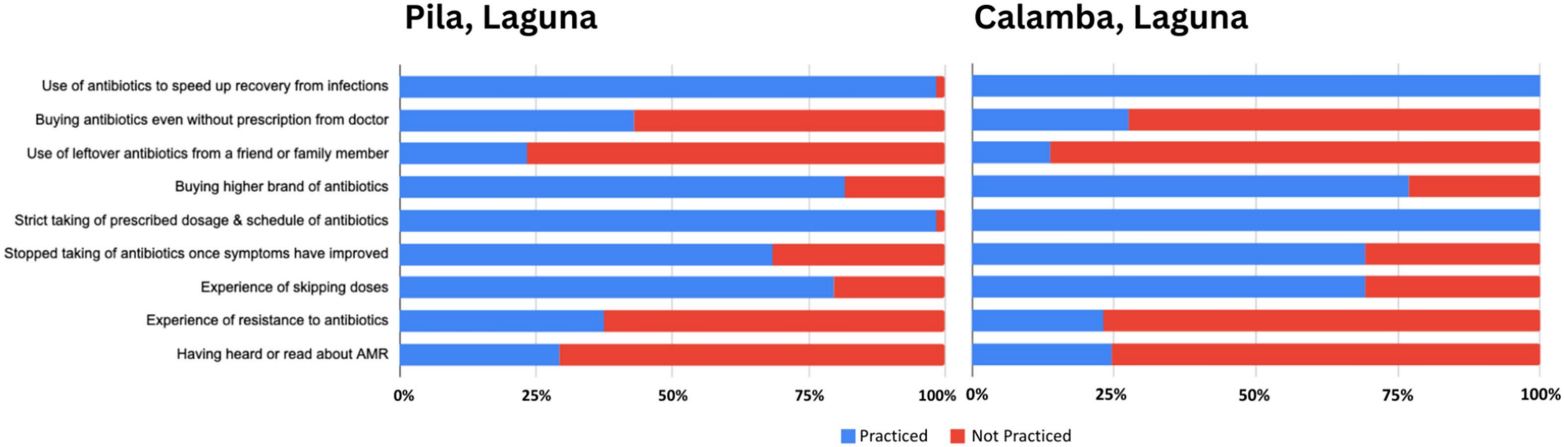
Comparative antibiotic use practices in Pila and Calamba, Laguna.

The following figures present comparative analyses of respondents’ knowledge (Figure 3), attitudes (Figure 4), and practices (Figure 5) regarding antibiotics, highlighting key differences that may contribute to shaping local health behaviors.

A comparative analysis of the survey data from Pila and Calamba, Laguna, reveals that while participants in both areas share a high foundational awareness that antibiotics are meant for infections (95.38% in Pila and 96.92% in Calamba), participants in both communities grapple with a dangerous “viral myth,” where over half wrongly believe these drugs treat viral illnesses (59.38 and 53.85%, respectively). Pila demonstrates slightly stronger adherence to the importance of following prescriptions (92.31%) compared to Calamba (84.62%); yet participants in Pila seem to more likely believe that treatment can be stopped prematurely once symptoms fade (66.15%) than those in Calamba (50.77%). A critical, shared vulnerability is the widespread unfamiliarity with the term “antibiotic resistance,” reported by a majority in both Pila (76.92%) and Calamba (70.31%). However, Calamba faces unique socioeconomic and cross-species misconceptions, such as the belief that brand-name price dictates efficacy (66.15%) and that human antibiotics are interchangeable for animal use (46.15%). This suggests that while Pila participants require interventions focused on treatment completion, Calamba participants necessitate broader education on drug quality and multi-use risks.

Analysis of attitudes toward antibiotics in Pila and Calamba, Laguna, reveals a shared baseline of high public trust in medication efficacy (95.38 and 98.46%, respectively) and medical authority (96.88 and 93.85%), yet participants in both communities exhibit a tendency to prematurely discontinue treatment once symptoms improve (66.15 and 63.08%). While Calamba participants demonstrate a more robust rejection of purchasing antibiotics without a prescription (76.92% disagreement) compared to Pila, where over a third of respondents (34.38%) find non-prescription purchases acceptable, participants in both areas struggle with the perceived simplicity of managing AMR. Pila participants display a higher degree of optimism regarding the ease of addressing AMR (57.81%) than Calamba participants (47.69%), although the latter exhibits some uncertainty (32.31%) on the topic. Ultimately, while slightly more Calamba participants show stronger adherence to formal medical channels regarding procurement, participants in both municipalities require targeted educational interventions to correct the persistent misconception that symptomatic relief equates to the completion of bacterial eradication.

Comparing antibiotic practices among participants in Pila and Calamba, data reveal that while both communities demonstrate a universal intent to use medication for recovery, they are plagued by high rates of non-compliance that jeopardize public health. Participants in both areas report a strong commitment to following prescribed dosages—though Calamba participants report a perfect adherence rate (100%) compared to Pila’s slightly lower but still high Figure (90.77%)—yet this is contradicted by the prevalent habit of skipping doses, which is more frequent in Pila (78.46%) than in Calamba (69.23%). Participants in both municipalities show an identical tendency to stop medication prematurely once symptoms improve (66.15% in Pila and 69.23% in Calamba). Nearly half of Pila participants buy antibiotics without a prescription (43.08%), whereas the majority in Calamba avoid this practice (72.31%). While Pila participants show a higher personal experience with resistance (36.92%), over three-quarters of Calamba participants have never heard of the term “antimicrobial resistance” (75.38%) compared to Pila respondents (29.23%).

- *Perception of branded versus generic antibiotics*

FGD participants in Calamba noted perceived differences in the efficacy of branded and generic antibiotics. Some participants mentioned that they recovered faster when they took branded antibiotics unlike when they took “*generic lang*” (generic only) wherein it took some time for them to recover from the symptoms of their illnesses. The use of the term “*generic lang*” alludes to the perception that generic drugs are inferior. One participant, however, reported the development of skin rashes after taking branded antibiotics in the past, which led to a present preference for generic alternatives.

- *Prevalence of Self-Medication and Reuse of Prescriptions*

Many FGD participants in both study sites reported self-medicating with antibiotics based on past experiences and reusing old prescriptions. This practice was particularly common in recurring conditions such as coughs and colds, urinary tract infections, and wound infections. In both communities, amoxicillin was the most popular antibiotic used, although other antibiotics were also used with less frequency. Reliance on personal experience contributes to frequent self-medication with antibiotics. One health worker in Calamba narrated that some of the individuals in her community self-medicated being unaware of the risks involved in this practice.

- *Fear of side effects*

More male than female FGD participants in both sites reported not completing the prescribed period for taking antibiotics. In Calamba, female participants demonstrated awareness that incomplete treatment could result in antibiotic resistance. In contrast, male participants in Pila, described modifying their dosing schedules, with some stopping after about 2 or 3 days or once symptoms subsided. One participant reported taking the medicine every other day or every 2 days, believing that spacing doses reduces the likelihood of suffering from side effects.

Fear of damaging the kidneys was mentioned as a possible side effect of taking too many antibiotics or taking antibiotics for an extended period. Seven days was considered a long duration for antibiotic intake. The experience of side effects, such as dizziness or digestive discomfort, frequently led participants to discontinue antibiotics or switch to herbal alternatives. Participants also noted that adverse side effects resulted in missed work and income.

- *Mode of intake of antibiotics*

The FGDs also revealed that it was a common practice for participants to break open a capsule or pulverize a tablet and sprinkle the powder over an open wound to prevent an infection. This was perceived as “safer” than ingesting the drug since this was only applied topically. One participant shared that she mixed the pulverized antibiotic with food such as fresh pineapples, in the belief that it helped in the fast healing of wounds from a caesarian section. A female FGD participant in Calamba said,

“*When I gave birth via cesarean section, I ate fresh pineapples with the antibiotics to ensure speedy recovery*.”

#### 3.2.2 Interpersonal level

- *Prescription sharing*

FGD participants reported using prescriptions intended for other family members or friends when experiencing similar symptoms. They reasoned that they could share the same illness since the symptoms were the same. Hence, instead of seeking medical attention, they would simply use the same prescription or share the same medicines being taken by the other afflicted family member. In Calamba, one female FGD participant claimed to have heard about antibiotics from her adult children and took these when she fell ill.

*“I just learned about the antibiotic I took from my children. They said that they learned about it from doctors when they were sick themselves.”*

#### 3.2.3 Organizational level

- *Purchasing antibiotics from the pharmacy without a prescription*

All the interviewed pharmacy staff from both communities indicated that they do not dispense antibiotics without a prescription. In instances wherein community members insist on purchasing without a prescription, they would educate the buyer on the dangers of taking antibiotics without proper consultation from a physician and of not completing the required duration of intake. In Calamba, pharmacy staff explained this risk saying that they will only make the antibiotic angry, implying that improper use would render the antibiotic ineffective the next time it is needed. In this instance, antibiotics are personified as a way of communicating the concept of resistance to customers. The Calamba pharmacy staff said,

“…*by not completing the prescribed treatment course, they are only making the antibiotic angry.”*

- *Brand preference of healthcare service providers*

The survey in both communities revealed that most respondents preferred branded antibiotics, which were believed to possess greater efficacy compared with generics especially for the more severe infections. The interviewed health service providers also had the same general impression. One health professional explained that he would prescribe branded antibiotics for his own family, relatives, and friends, noting that patients tend to be more “hiyang” (compatible) with them and therefore experience fewer side effects, although he acknowledged that generics and branded antibiotics should have the same efficacy. The concept of “compatibility” with specific medicines or brands of medicines was discussed by Whyte et al. (20). This finding should not be understood simply as a preference for branded medicines. Rather, the notion of *hiyang* reflects a culturally grounded interpretation of how medicines interact with the body, where the absence of side effects and the speed of perceived recovery are taken as evidence of compatibility, while discomfort or slower improvement is interpreted as incompatibility. This interpretation directly shapes treatment-taking behavior. When patients feel that a medicine is not *hiyang*, they may discontinue antibiotics prematurely, shift to another brand without medical guidance, or seek alternative forms of treatment. In this sense, the concept of “compatibility” provides an analytical explanation for treatment non-adherence rather than merely describing patient preference. When applied to antibiotic use, these practices can result in incomplete or inconsistent antibiotic exposure, which creates conditions that contribute to the emergence of AMR.

- *Integration of Formal and Informal Healthcare Networks*

The FGDs show that participants are inclined to self-medicate first before seeking the help of health professionals. When they do not feel better after self-medication, they will go to traditional health practitioners; if they still feel that they are not getting well, only then would they seek professional medical health advice. FGDs in all sites refer to their preference for following long- held health beliefs passed down by elders. They typically wait for 2 to 3 days, and if their condition does not improve, only then would they go to either the folk healer, who was usually also a respected member of their community, or visit the health center for consultation.

Traditional healers are popular among community members, especially in Pila. The distance from certain barangays to the Rural Health Unit posed a significant deterrent for regular check-ups and medical consultations among their residents. For community members residing in Unserved/Underserved Areas (UUA) transportation costs can be high. Moreover, the time lost to travel also meant income loss as they would have to skip work and lose income. Hence, they would seek the advice of traditional healers who are usually within walking distance from their residences. The health professional in Pila mentioned that he knew of some traditional healers or *herbolarios* who also “prescribe” antibiotics. Aside from herbal remedies for various ailments, some *herbolarios* would also write down the names of certain drugs that could be bought from pharmacies as some sort of “prescription.” He noted one such “prescription” for Augmentin, which one of the patients in the health center showed him. He said,

“*Some time ago, I saw a “prescription” issued by an “herbolario.” It’s good that this was not honored by the pharmacy. I thought it was funny and I was surprised at the same time because the “prescription” named an antibiotic brand.”*

#### 3.2.4 Community level

- *Antibiotics bought from community variety stores*

Although pharmacy staff claim that they do not dispense antibiotics without a doctor’s prescription, community members can still purchase them from community variety stores (*sarisari* stores). In both rural and urban sites, FGD participants mentioned resorting to taking what they called as the “*mag asawang gamot*” (coupled medicine), which is composed of an antibiotic (Amoxicillin) and paracetamol (Biogesic). This combination of drugs can be bought prepacked in a small plastic bag from community variety stores without a prescription. It was generally believed that when taken together, these medications can cure any malady. Hence, they are taken not only when an individual is suffering from an illness but also when feeling exhausted particularly when men return home from a day’s work, especially those employed as casual laborers in construction sites.

- *Use of antibiotics for animals*

Antibiotics were also freely used with farm animals and pets. Farm animals, such as chickens, are usually raised to serve as food for the family when they reach the appropriate age and size. Chickens and dogs were given antibiotics for colds or when they were *matamlay* (lacking energy). Pharmacy staff claimed that even if the purchased antibiotic would be used for animals, they still ask for a prescription before selling it. A pharmacy staff from Pila, Laguna mentioned that in one instance, a buyer used an old prescription intended for humans to purchase antibiotics for his roosters.

The FGD participants in both sites mentioned that antibiotics formulated for humans are also safe and effective for pets and farm animals. They also mentioned that unused antibiotics are often given to these animals when needed. A female FGD participant from Pila narrated that,

*“I saw my neighbor give antibiotics intended for humans to his pet kitten. The kitten was cured.”*

One participant in Pila, however, mentioned that she used only antibiotics for animals, such as *Vetracin,* to treat her farm animals (i.e., chicks). She stated that antibiotics for humans are more costly so it would be a waste of money to use them on farm animals. She does not follow a specific regimen in treating her chicks; she mentioned that she just mixes whatever amount she deems sufficient into the water she provides them.

- *Cultural beliefs on the healing properties of Laguna Lake*

In Pila, participants shared a common belief that Laguna Lake possesses healing properties. Hence, whenever children became sick, they were brought to the lake to heal. It was generally believed that the waters of the lake, especially at a point far from the shore where the water is clear, could heal a child who inhaled the clean air and/or was dipped in its waters. One FGD participant recounted bringing her daughter, who had severe coughs and colds, to the lake. After being dipped in the water, her daughter coughed up profuse mucus, experienced relief from symptoms and eventually recovered.

#### 3.2.5 Institutional (policy) level

Apart from the “no prescription, no dispensing” policy articulated by the pharmacy sales assistants, no other AMR-related policies were mentioned by the research participants. Existing programs to increase awareness of AMR and ensure responsible antibiotic stewardship were not mentioned.

Only one of the pharmacy assistants interviewed has undergone formal training in pharmacy services. The pharmacy assistant in Calamba held a Pharmacy Services NC III certificate. TESDA is a government agency that offers a competency-based certification program aimed at professionalizing pharmacy assistants. Only one of the pharmacy assistants interviewed has completed this training. Pharmacy sales assistants perform an important role in antimicrobial stewardship, and training on the proper and judicious dispensing of medicines, such as antibiotics, is essential to fulfilling this role.

In the study sites, the BHWs were respected and recognized by community members for their knowledge in health matters. They were often consulted for their health concerns, including medications like antibiotics. They also serve as liaisons between the health center and the community members about available health services and schedules. In this study, formal training for the BHWs seems to be few and far between. Nonetheless, they felt the need to upgrade their knowledge and skills and expressed willingness to spend time attending training.

## 4 Discussion

In one of the study’s field sites, residents engage in corporeal cleansing and sun-drying as an alternative premedication practice for illness prevention and treatment. Given their proximity to Laguna Lake, they have direct access to its waters, which they use for bathing before allowing their bodies to dry under the sun—a process reminiscent of traditional fish-drying techniques. This practice is based on the belief that the lake’s water possesses cleansing properties, while sunlight serves as a natural sterilizer against bacteria and viruses. These beliefs, which have been passed down through generations, are deeply embedded in local folk-medical traditions, shaping how individuals negotiate healthcare access within the constraints of a struggling economy. The folk-medical practice mentioned suggests that health is not merely a biomedical issue but a deeply social one, shaped by historical, cultural, and economic contexts.

Recognizing these conditions, this study underscores a critical yet often overlooked perspective in medical discourse. As Geiger (21) observed, medical issues are inherently social issues and can only be effectively addressed when communities are informed, engaged, and actively involved in crafting solutions. By examining these local health practices, this research highlights the importance of community collaboration in public health interventions and advocates for inclusive, context-sensitive approaches to healthcare delivery.

The study shows the relevance of using the SEM in understanding behaviors surrounding AMR in various community contexts. It also points to the need for the development of a diverse set of strategies designed to combat AMR by revealing the intricacies of the ecological and social systems to gain better insights for the design of interventions for an AMR resilient community (18). Lambraki, et al. (17) have identified the value of building adaptive socio-ecological systems to address the challenge of AMR. Wernli et al. (22) emphasized the importance of delving into the particular political, economic, cultural and ecological contexts that drive AMR behavior in different socio-political, economic and ecological settings for the design of resilient AMR governance mechanisms.

### 4.1 Going beyond individual behavior

Antibiotic use is shaped by socio-ecological factors that influence health-seeking behaviors. The study has shown that looking at both the individual and the broader context of the individual was useful in identifying significant factors affecting antibiotic resistance. Examining the broader contexts that influence individual behavior allows insights that could inform the development of more targeted strategies that address not only individual behavior, but also structural inequalities affecting antibiotic use and misuse. Poirier et al. (23) emphasize the need for greater engagement with social science perspectives to accelerate national AMR initiatives. This requires policy provisions that would be able to address structural inequalities that serve as barriers to rational antibiotic use that emanate from the broader environment of the individual. The PNAP for combatting AMR, in its several iterations, from the initial PNAP covering the years 2015 to 2017 to the more recent version covering 2024 to 2028, has consistently argued for a holistic perspective in addressing AMR through the One Health lens (24). One Health is an integrated approach that recognizes that the health of people, animals and the environment are intertwined. This approach calls for the need for transdisciplinary perspectives in the development of strategies and programs to address AMR.

### 4.2 Bridging the gap in knowledge, attitudes and practices

The KAP survey conducted in this study revealed that knowledge of completing the prescribed treatment regimen requiring antibiotics was high with survey results reaching over 90 percent in Pila and above 86 percent in Calamba. However, the survey results also show that there is inconsistency between knowledge, attitudes and practices among the participants, particularly in following the complete course of prescribed treatment. Similar findings were also found among Saudi Arabia residents (25). Most of the participants in this study felt that it was adequate to discontinue treatment once the symptoms subside. The findings from the survey support findings from the FGDs wherein some participants confirmed not completing the prescription, citing fear of side effects, especially with long-term use of antibiotics and the perception that they were cured of the illness since the symptoms have already subsided. Mallah et al. (26) noted that approximately 29–95% adults globally held misconceptions about the indications, instructions for use, and side effects of antibiotics. These misconceptions, documented across different countries, point to the need for more aggressive educational campaigns. This study has also shown, however, that educational campaigns must be supported by interventions that address other factors that encourage behaviors that counter the correct knowledge about antibiotic use. Educational interventions alone may not be sufficient to mitigate the growing concern over AMR.

### 4.3 Gendered practices

The study findings show that women, more than the men, tended to comply with prescribed treatment. This finding is consistent with the findings of another study conducted in San Pablo City, in the same province of Laguna (27). Women’s role as healthcare gatekeepers in their homes and in their communities have been documented in other cultural settings (28, 29). Studies have shown that women are the ones responsible for making healthcare decisions for the family. In the FGDs done in the study sites, the men acknowledged this primary role of the matriarch in their homes. Health programs aimed at mitigating AMR in communities may strengthen the role of women therein as drivers of health practices in the home and their communities. It is also important to ensure women’s health so they can effectively carry out this role.

### 4.4 Negotiating “side” effects

FGD participants in Pila, especially men, mentioned that they tended not to complete the prescribed course of treatment, often stopping medication once symptoms subsided or spacing their intake of antibiotics due to the “side” effects they experienced. Moreover, the concept of “*hiyang*” was also mentioned in the FGDs wherein side effects were more likely to be encountered if the patient was not “*hiyang*” with the medicine or a specific antibiotic brand. While “*hiyang*” is a Filipino term, there are similar concepts elsewhere in the Philippines and in other ASEAN cultural contexts as well (30). Fear of side effects and their consequences—such as downtime resulting in missed work and income—were a greater concern among male than female FGD participants. In this instance, side effects are negotiated as a response to the adverse side effects experienced. Negotiating side effects was also reported by Etkin (31) in her study among the Hausa people in Northern Nigeria.

### 4.5 Medical pluralism and medical syncretism

Health-seeking behavior of the study participants reflected Kleinman’s (32) plurality of medical systems through his three-sector scheme. This included the popular, folk, and professional health care sectors, which are distinct yet have fluid boundaries. It is therefore important to understand local beliefs and practices.

Self-medication was practiced in both sites, whereby participants took medicines, including antibiotics, previously prescribed for common ailments such as headaches and open wounds deemed not serious enough to require medical attention. Self-medication for common aches and pains was also a common practice in Tanzania (33) and elsewhere in the Philippines (11, 34). For recurring illnesses such as UTIs, some FGD participants claimed that they would forgo medical consultation and proceed with taking previously prescribed antibiotics, recycling prescriptions issued to them in the past. Traditional healers were also consulted because they were more accessible. Many traditional healers lived within the communities of the research participants, often within walking distance from their homes, and were well respected as healthcare providers (35). Doing so would allow people to save on travel time and expenses. Interestingly, one KI mentioned that he knew of a traditional healer who also issued “prescription” medicines, including antibiotics. It can be inferred that with the practice of dispensing prescriptions, traditional healers are able to give the impression of “professionalism,” hence further uplifting their authority as healers in their communities. Many of the research participants claimed that the professional medical sector was not easily accessible and was often the last resort when both the popular and the traditional medical sectors failed. However, at this stage, the disease may be well in its advanced stage. Medical syncretism, as described by Kleinman (32), makes it easier for people in societies characterized by health sector heterogeneity to switch between providers. Thus, it appears that it is imperative for professional medical care to be made available not only geographically but also economically.

Popular medical beliefs and practices may also be linked to broader environmental beliefs, reflecting and reinforcing a socio-ecological perspective on health and wellbeing. Treatment regimens reflect views about illnesses and their etiologies. This study showed that participants also held a mystical view of Laguna Lake, along with healing beliefs and practices. Excursions to the lake and dipping in its waters were mentioned by research participants as a popular practice to heal common coughs and colds, especially among children. This was commonly reported by Pila participants as an alternative to seeking medical attention and advice from health professionals. Coughs and colds, unless symptoms of more serious conditions, are often self-limiting. It is possible that recovery from common coughs and colds after a dip in Laguna Lake has been attributed to the healing properties of the Lake rather than to a self-limiting infection. Cultural beliefs that inform health-seeking behavior also shape treatment choices, with traditional healers often sought as a first line of care. In such instances, more serious conditions may not be diagnosed, and appropriate treatment may be missed. The literature points to several lakes with similar mystical healing properties. For instance, Lake Titicaca on the Peru-Bolivia border figured prominently in the literature in relation to pilgrimage and healing (36). Lake Atitlan in Guatemala has already been viewed by indigenous lakeshore communities as inextricably linked to their cultural identity (37). In the Philippines, Lake Sebu figures prominently in Maranao beliefs and healing rituals (38). In Batangas, Peracullo (39) documented narratives on the therapeutic properties of Taal Lake in relation to people’s devotion to the Our Lady of Caysasay. The Lady is a popular Marian image, especially in narratives on miraculous healing.

### 4.6 Non-professional access pathways to antibiotics

Although pharmacy assistants mentioned that they strictly adhere to government regulations regarding dispensing of antibiotics, such as requiring the presentation of a current prescription and filling only the prescription presented, antibiotics may still be freely accessed through other pathways. Easy access through non-professional routes may not be covered by government regulations and policies. Using the SEM lens reveals such information, which may help broaden the scope of and strengthen regulations and policies on dispensing medicines.

Non-completion of prescription was reported as a practice by participants of the FGDs at both study sites. Economic constraints, accessibility challenges, and experiences of adverse effects often lead individuals to abandon antibiotic treatment in favor of traditional remedies, further exacerbating risks associated with AMR. Prescriptions issued by medical doctors indicate the total amount of antibiotics needed for the full course of treatment. The government health centers also provide antibiotics sufficient for one full treatment period. When patients purchase the total amount of antibiotics but do not complete the full course of treatment, the remaining medicines are not discarded, leaving patients with excess antibiotics at home. Usually, these are stored in a medicine cabinet or any part of the house with easy access by household members. The next time a family member experiences the same symptoms, they may easily access these medicines even without seeking professional medical advice. Household storage of medicines was also observed in Costa Rica (40) and was found to increase health risks because easy access led to unguided use. This finding underscores the importance of ensuring that medical advice also incorporates proper disposal of excess medicines.

Moreover, it is not only the pharmacies that sell antibiotics. *Sarisari* stores are not only sources of antibiotics but also provide the *mag-asawang gamot,* which participants claim to be a cure-all and are often consumed even for simple muscle aches, pains or general body malaise. These are easily affordable, as they are sold as a bundle composed of one antibiotic and one paracetamol.

### 4.7 Off-label use of antibiotics

- *In Animals*

Off-label use of antibiotics was also reported by some participants. There were occurrences in which leftover antibiotics prescribed for humans were administered to livestock or poultry raised for household consumption. This practice increases the risk of AMR among family members who consume livestock. Furthermore, leftover medications previously used and found effective in humans were often given to domesticated animals when they appeared sluggish or lethargic, which was perceived as a sign that they were unwell. This practice was carried out instead of spending limited household financial resources to purchase new veterinary medicine. In many cases, dosages were empirically estimated rather than determined according to veterinary prescriptions, which are computed based on the actual physiological and health requirements of the animals. With such practices, the danger of overdosage and underdosage is possible. Thus, the indiscriminate use of antibiotics increases the risk of AMR not only in animals but also among humans who handle them. The use of antimicrobials in backyard farms in the Philippines and its relation to AMR risk was studied by Barroga et al. (47). The expansion of AMR surveillance points beyond human and commercial livestock use may be incorporated into national-level policy.

- *In Humans*

The participants in the FGDs reported their adjustments on the use of antibiotics depending on the medical condition, often without the guidance from a healthcare professional, representing a form of off-label use. For example, illnesses were generally perceived as requiring antibiotic treatment. In the case of wounds, which are external medical conditions, some participants felt that applying pulverized antibiotic tablets directly to wounds was as effective as ingesting them. They also felt that this mode of application lessened side effects. Some participants also mentioned that they would mix the pulverized antibiotic with pineapple juice. Doing so masked the unpalatable taste of the antibiotic. However, because these practices were not in accordance with prescribed methods, they may result in decreased potency and efficacy of the antibiotic.

### 4.8 Symbolic value of antibiotics

Antibiotics, like other medicines, hold symbolic value beyond their curative functions. The authority of prescriptions as a legitimizing and effective tool was also described by Chandler et al. (41) as an important dimension of health care. The prescription allows access to medicines, especially those that cannot be obtained over the counter like antibiotics. It also imbues the prescribing individual with authority and legitimizes his or her role as a knowledgeable health care provider. In the study sites, some traditional healers dispense prescriptions, which could have enhanced their role as health care providers in the community.

Patients view antibiotics as a cure-all for various aches and pains. This view encourages the indiscriminate use of antibiotics. Moreover, there was a preference for branded medicines, including antibiotics, based on the view that more costly branded medicines were more efficacious compared with the generics. Such a view is not limited to non-health professionals. In this study, a health professional also articulated this view. Such beliefs may contribute to individuals abandoning treatment thinking that they are being prescribed an inferior treatment regimen. In India, Uzair and Siddiq (42) likewise found that this view persisted among medical doctors, even when they were aware of the clinical equivalence between branded and generic antibiotics.

### 4.9 Continuing education for first line health care workers

First-line treatment for health concerns is often sought from pharmacy sales assistants and the BHWs. Hence, continuing education to upgrade their knowledge and skills is essential for them to continue to perform their roles effectively. Republic Act No. 10918, or the Philippine Pharmacy Act, requires all persons handling pharmaceutical products, including pharmacy sales assistants who are part of the pharmacy support workforce, to undergo accreditation training programs. Undergoing these trainings ensure that pharmacies employ responsible pharmacy assistants who can competently manage the demands of their jobs. BHWs are also covered by Republic Act No. 7883, which has provisions for professionalization through certification by local health boards and a Department of Health prescribed training program.

### 4.10 Strengths and limitations

The study utilized the survey, FGD and KII methods of data collection thus allowing triangulation of data to enhance validity and credibility of the findings. Moreover, the SEM in which this study was framed as well as covering both an urban site and a rural site, may provide a broader view of varying contexts that may shape behaviors and decision making pertinent to AMR. The inclusion of health workers and pharmacy assistants as well as end users of antibiotics provide a wide range of AMR perspectives.

A limited view of the health professional’s perspective was provided since only one health professional was covered. However, this does not undermine the overall comparative analysis. Triangulation across KAP surveys, FGDs, and key informant interviews including pharmacy assistants and Barangay Health Workers provided sufficient insight into community-level knowledge, attitudes, and practices regarding antibiotics allowing a comparison of the socio-ecological drivers of antibiotic use and AMR between the two sites.

Also, non-probability sampling design was employed which delimits the applicability of the study findings to lakeshore communities in Laguna. While the results are context-specific, similar approaches may be relevant in other settings where communities experience comparable social and structural conditions.

## 5 Conclusion and recommendations

AMR is a complex issue that requires a holistic approach to mitigate. This study has shown that there are social, cultural, economic and regulatory issues that need to be acknowledged and addressed. Situating antimicrobial use within its broader socio-ecological context is evident in this study. Interventions that are informed by a deep understanding of folk, traditional, and professional medical healthcare-seeking patterns are warranted. The findings highlight the need for collaborative community-centered interventions that integrate local knowledge and address structural barriers to antimicrobial use in lakeshore communities in Laguna.

Practices at the community level revealed limited implementation of existing policies. For instance, in this study, antibiotics may be bought from community variety stores. While the Philippine Pharmacy Act restricts the sale of antibiotics to only licensed pharmacies, antibiotics are sold in community *sarisari* stores in the study sites. Regulatory agencies have to be made aware of the existence of unregistered sources of antibiotics and to exercise greater vigilance to ensure that regulations are followed in the sale of antibiotics at all points.

Increasing awareness of and enforcement of AMR policies requires a whole of society approach. Health concerns are not the responsibility of the health sector alone. The engagement of multiple sectors in society is necessary to achieve greater awareness and improve compliance. The role of the education sector in improving health literacy through the school curricula is warranted. For instance, the *OK (Oplan Kalusugan) sa DepEd*, a health program of the Department of Education may incorporate AMR as one of its core components so even elementary level learners are already informed of the risks and importance of preventing AMR. Community-based education programs on AMR through integration of messages in routine health education campaigns may help improve community awareness. Popular and traditional communication platforms may be utilized to further boost awareness and reach of key AMR messages. Social media has become increasingly popular among communities. Social listening systems may be adopted by the health sector in monitoring messages that are being disseminated and who are reached by these messages. The Local Government Units can be powerful agents in ensuring that policies are disseminated and enforced. The PNAP has already identified the One Health lens as a means through which collaborative and multisectoral programs to combat AMR can be designed. Addressing the social inequalities that drive AMR through the PNAP requires the involvement of other government agencies within the ICAMR such as the Department of Social Welfare and Development, for instance to ensure that equitable support for AMR prevention is provided.

Informal healthcare networks function as adaptive responses to systemic challenges, including financial constraints, limited access to healthcare facilities, and inadequate healthcare infrastructure. These networks may offer immediate but at times, inconsistent solutions to health concerns. While these informal strategies provide essential healthcare alternatives, they may also contribute to the improper use of antibiotics, thus, increasing public health risks. Enhancing access to affordable healthcare, especially in the areas which are distanced from the main health center or with lack of healthcare facilities, is imperative. Health centers may conduct regular medical visits by the health personnel who provide medical consultation services and bring medicines, vaccines and portable diagnostic medical devices to UUAs.

The presence of informal healthcare networks is further compounded by misinformation and gaps in health literacy. Many community members depend on advice from peers, often leading to incorrect diagnoses and inappropriate antibiotic use. This gap in health literacy extends to AMR, where the misuse of antibiotics accelerates the development of drug-resistant infections, posing long-term risks to public health. Addressing these issues requires targeted health literacy initiatives that are grounded in local contexts, ensuring the dissemination of accurate and culturally relevant information about antibiotic use and resistance. Strengthening health literacy can, in turn, mitigate reliance on informal healthcare sources and reduce the prevalence of AMR.

At the same time, many communities integrate traditional remedies, such as herbal medicine, alongside formal medical interventions, reflecting deeply embedded cultural knowledge. The availability of medicinal plants and other ecological resources further influences treatment choices, illustrating the interconnections between healthcare practices and local environments. Recognizing these cultural and ecological dimensions is essential for designing health interventions that are both evidence-based and socially accepted. By integrating traditional knowledge with modern healthcare systems, public health initiatives can enhance community engagement, improve health outcomes, and contribute to more sustainable healthcare strategies.

Pharmacy sales assistants and BHWs are frontline healthcare providers. While they are often sought for advice on antibiotic use, their role in antibiotic stewardship remains hidden. Recognizing their role therein may provide the necessary push for the professionalization of their occupations so that opportunities are made available for them to upgrade their knowledge and skills as they undertake their tasks within the bounds of their occupations. To allow this, more stringent enforcement of the Philippine Pharmacy Act and RA 7883 for BHWs is deemed to be urgent and necessary. Moreover, the ratification and implementation of the Magna Carta of Barangay Health Workers are vital to tackle the inadequacy of regulatory frameworks that cater to the needs of BHWs.

Studies on the socio-ecological context of AMR across a range of social and cultural settings are necessary for fine tuning of intervention. Moreover, this study has revealed that gender is a salient driver of practices related to AMR. More in-depth studies that look into gender norms and intersections with other dimensions of social life is warranted to understand better gender dynamics in the context of AMR. The study also showed that women were actual gatekeepers of health in their homes, therefore, studies on avenues and mechanisms within the household and the community to further empower the role of women as health gatekeepers and AMR prevention advocates can be explored.

## Data Availability

The raw data supporting the conclusions of this article will be made available by the authors, without undue reservation.
