# Supplementary material for: Using the socio-ecological model in understanding antimicrobial resistance and antibiotic usage in the lakeshore communities of Calamba and Pila, Laguna, Philippines
Source: Front Public Health. 2026 May 11;14:1827837. doi: 10.3389/fpubh.2026.1827837 (PMC13199311; doi:10.3389/fpubh.2026.1827837)
Supplement: Supplementary file 1 [file Data_Sheet_1.PDF]

Combating Antibiotic Resistance in Philippine Lakes: One Health upstream interventions to reduce the burden (ARPHILAKE)

KAP Survey Questionnaire
English and Filipino Version & date: March 11, 2024

**Unang Bahagi: Mga Sosyo-Demograpikong Katangian at Paggamit ng Antibiotics /** Part 1: Socio-Demographic Characteristics and Antibiotic Use			
**Petsa kung kailan isinagawa ang panayam** / Date questionnaire was accomplished:			
**Pangalan ng Tagapanayam** / Name of Interviewer:			
**Uminom ka na ba o kasalukuyang umiinom ng antibiotics?** / Have you taken or are currently taking antibiotics?	☐ **Oo** / Yes ☐ **Hindi** / No		
**Pangalan ng Kinapanayam (Opsyunal)** / Name of Respondent (Optional)			
**Petsa ng Kapanganakan (bb/aa/taon)** Date of Birth (mm/dd/yyyy)		**Taong Gulang** / Age	
**Tirahan** / Residence **(Barangay, Lungsod)** / (Barangay, City)			
**Relihiyon /** Religion			
**Numero ng Telepono** / Mobile			

Number	
**Kasarian na itinakda sa kapanganakan** / Sex assigned at birth	☐ **Lalake** / Male ☐ **Babae** / Female ☐ **Iba pa** / Others: ________________<br>☐ **Hindi nais na sagutin** / Prefer not to answer
**Pinakamataas na natapos** / Highest Education	☐ Primary (Elementarya) / Primarya (Elementarya)<br>☐ Secondary (High School) / Sekundarya (Hayskul)<br>☐ Technical-Vocational / Teknikal-Bokasyunal<br>☐ Tertiary (College) / Tersyarya (Kolehiyo)<br>☐ Post-Graduate / Higit pa sa gradwadong pag-aaral<br>☐ Other / Iba pa:___________________
**Trabaho** / Occupation	
**Buwanang sahod** / Monthly Income	☐ **Mababa sa** / Below P10,957.00<br>☐ P10,957.00 - P21,913.00<br>☐ P21,914.00 - P43,827.00<br>☐ P43,828.00 - P76,698.00<br>☐ P76,699.00 - P131,483.00<br>☐ P131,484.00 - P219,139.00<br>☐ Mataas pa sa P219,139.00<br>☐ **Walang trabaho** / Not employed<br>☐ **Hindi naaangkop** / Not applicable
**Ano ang mga pinagkukuhanan mo ng impormasyon tungkol sa antibiotics?** / What are your sources of information on antibiotics?	☐ **Tagapangalaga ng kalusugan, sino?** / Health care provider (HCP), who?<br>_________________________<br><br>☐ **Kapit-bahay, kamag-anak, kaibigan, katrabaho /** Neighbors, family members, friends, co-workers<br>☐ **Albularyo** / traditional healers<br><br>☐ **Social Media, mangyaring pakitukoy?** / Social Media, which?<br>____________________________________<br><br>☐ **Dyaryo** / Newspapers<br><br>☐ **Telebisyon** / Television<br><br>☐ **Radyo** / Radio

	☐ **Anunsyo sa komunidad (ex, bandilyo)** / Community announcements ___________________<br><br>☐ **Iba pa, mangyaring pakitukoy** / Others, please specify ____________________________________

**Ikalawang Bahagi: Kaalaman sa Paggamit ng Antibiotic at Antibiotic Resistance / Part 2: Knowledge on Antibiotic use and Antibiotic Resistance**	
**Maaari bang gamutin ng antibiotics ang mga impeksyon? /** Can antibiotics cure infections?	☐ **Hindi** / No ☐ **Hindi ko alam** / Undecided ☐ **Oo** / Yes<br>**Kung ang sagot ay "Oo," sagutin ang mga sumusunod na katanungan.** / If answered "Yes," answer the following questions.<br>1) **Alin sa mga sumusunod na impeksyon ang maaaring gamutin ng antibiotics? (Markahan ang lahat ng naangkop na kasagutan.)** / Which of the following infections can be cured by antibiotics? (Mark all that apply.)<br><br>☐ **Ubo at sipon** / coughs and colds<br>☐ **Sakit sa lalamunan** / sore throat<br>☐ **Sakit sa balat** / skin diseases<br>☐ **Impeksyon sa pag-ihi** / UTI (Urinary Tract Infection)<br>☐ **Problema sa ngipin hal. pagtanggal ng ngipin, impeksyon** / Dental health issues ex. tooth extraction, infections<br>☐ **Problema sa Obaryo** / Ovary (cesarean section)<br>☐ **Mga sugat** / Open wound<br>☐ **Matapos operahan** / Post-surgery<br>☐ Others / iba pa: ____
**Maaari bang gamutin ng antibiotics ang mga impeksyong dulot ng virus? /** Can antibiotics cure viral infections?	☐ **Oo** / Yes ☐ **Hindi** / No<br>☐ **Hindi ko alam o sigurado** / I don't know or I am uncertain
**Maaari bang mapabilis ng antibiotics ang paggaling mula sa mga impeksyon? /** Can antibiotics speed up recovery from infections?	☐ **Oo** / Yes ☐ **Hindi** / No<br>☐ **Hindi ko alam o sigurado** / I don't know or I am uncertain
**Maaari bang bumili ng antibiotics nang walang reseta mula sa doktor?/** Can antibiotics be	☐ **Oo** / Yes ☐ **Hindi** / No<br>☐ **Hindi ko alam o sigurado** / I don't know or I am uncertain

bought without prescription from the doctor?	
**Sa palagay mo, ang madalas bang paggamit ng antibiotics ay makapagpapapabawas ng bisa nito sa mga susunod na paggamit?** / Do you think frequent use of antibiotics will reduce its efficacy in future uses?	☐ **Oo** / Yes ☐ **Hindi** / No<br>☐ **Hindi ko alam o sigurado** / I don't know or I am uncertain
**Higit bang mabisa ang mahal na antibiotics kumpara sa mura?** / Is the efficacy of higher priced antibiotics better compared with lower priced brands?	☐ **Oo** / Yes ☐ **Hindi** / No<br>☐ **Hindi ko alam o sigurado** / I don't know or I am uncertain<br><br>**Bakit mo ito nasabi?** / Why do you say so? ___________________________
**Dapat bang sundin nang maigi ang pag-inom ng antibiotics ayon sa niresta?**/ Should antibiotics be taken strictly as prescribed?	☐ **Oo** / Yes ☐ **Hindi** / No<br>☐ **Hindi ko alam o sigurado** / I don't know or I am uncertain<br><br>**Kailan ka maaaring hindi sumunod o tumigil sa pag-inom ng gamot?** / When can you deviate or stop use as prescribed? /
**Maaari mo bang itigil na ang pag-inom ng antibiotics kapag bumuti na ang iyong mga sintomas?** / Can you stop taking antibiotics when your symptoms have improved?	☐ **Oo** / Yes ☐ **Hindi** / No<br>☐ **Hindi ko alam o sigurado** / I don't know or I am uncertain<br><br>**Bakit?** / Why?
**Maaari bang makaapekto o makasama ang sobrang dosis sa pag-inom ng**	☐ **Oo** / Yes ☐ **Hindi** / No<br>☐ **Hindi ko alam o sigurado** / I don't know or I am uncertain

**antibiotics?** / Can you suffer from overdose or side effects when taking antibiotics?	**Kung oo, ano ang mangyayari kung masobrahan sa dosis sa pag-inom? Ano ang mga epekto?** / If yes, what will happen if you overdose? What are the side effects?
**Maaari bang ang antibiotics na nasa mga hayop ay maipasa sa tao kapag kinain ang mga ito?** / Can antibiotics in animals be transferred to humans when they are eaten?	☐ **Oo** / Yes ☐ **Hindi** / No<br>☐ **Hindi ko alam o sigurado** / I don't know or I am uncertain
**Maaari bang mainom ang antibiotics kapag naglalangoy sa tubig na kontaminado nito?** / Can antibiotics be ingested when swimming in antibiotic-contaminated waters?	☐ **Oo** / Yes ☐ **Hindi** / No<br>☐ **Hindi ko alam o sigurado** / I don't know or I am uncertain
**Maaari bang gamitin ang antibiotics na para sa tao sa paggamot ng mga hayop?** / Can antibiotics meant for humans be used in treating animals?	☐ **Oo** / Yes ☐ **Hindi** / No<br>☐ **Hindi ko alam o sigurado** / I don't know or I am uncertain
**Narinig mo na ba ang antibiotic resistance o ang paghina ng bisa ng antibiotic?** / Have you heard of antibiotic resistance?	☐ **Oo** / Yes ☐ **Hindi** / No<br>**Kung oo, maaari mo bang sabihin sa akin kung ano ito?** If yes, can you tell me what this is about? ______________________

**Ikatlong Bahagi: Pananaw ukol sa Antibiotics at Antibiotic Resistance /** Part 3: Attitudes toward Antibiotics and Antibiotic Resistance			
**Ligtas inumin ang antibiotics.** / Antibiotics are safe. *Conditional yes	☐ Hindi sumasang-ayon / Disagree	☐ Hindi ko alam / Undecided	☐ Sumasang-ayon / Agree
**Epektibo ang antibiotics laban sa mga impeksyon.** / Antibiotics are effective against infections.	☐ Hindi sumasang-ayon / Disagree	☐ Hindi ko alam / Undecided	☐ Sumasang-ayon / Agree
**Maaaring ihinto ang paggamit ng antibiotics kapag bumuti na ang mga sintomas o pakiramdam.** / Use of antibiotics can be stopped once symptoms have improved.	☐ Hindi sumasang-ayon / Disagree	☐ Hindi ko alam / Undecided	☐ Sumasang-ayon / Agree
**Maaaring gumamit ng antibiotics ayon sa mga payo ng mga kapit-bahay, kaibigan,, o mga kamag-anak**. / It is ok to use antibiotics based on the recommendation of a neighbor, friend, or other family members.	☐ Hindi sumasang-ayon / Disagree	☐ Hindi ko alam / Undecided	☐ Sumasang-ayon / Agree
**Maaaring gumamit ng antibiotics ayon sa mga payo ng albularyo.** / It is ok to use antibiotics based on the recommendation of traditional healers.	☐ Hindi sumasang-ayon / Disagree	☐ Hindi ko alam / Undecided	☐ Sumasang-ayon / Agree
**Maaaring bumili ng antibiotics kahit walang reseta mula sa doktor.** / It is alright to buy antibiotics even without prescription from the doctor.	☐ Hindi sumasang-ayon / Disagree	☐ Hindi ko alam / Undecided	☐ Sumasang-ayon / Agree
**Hihimukin ko ang aking mga kamag-anak na gumamit ng antibiotics kapag kinakailangan**. / I would encourage my family members to use antibiotics when needed.	☐ Hindi sumasang-ayon / Disagree	☐ Hindi ko alam / Undecided	☐ Sumasang-ayon / Agree
**Ang mga doktor ang pinakamahusay na pinagmumulan ng impormasyon tungkol sa antibiotics.** / The doctors are the best source of information on antibiotics.	☐ Hindi sumasang-ayon / Disagree	☐ Hindi ko alam / Undecided	☐ Sumasang-ayon / Agree
**Ang antibiotic resistance o paghina ng bisa ng antibiotic ay madaling tugunan.** / Antibiotic resistance can be easily addressed.	☐ Hindi sumasang-ayon / Disagree	☐ Hindi ko alam / Undecided	☐ Sumasang-ayon / Agree

**Ikaapat na bahagi: Mga Pamamaraan sa Paggamit ng Antibiotics at Antibiotic Resistance /** Part 4: Practices on Antibiotic use and Antibiotic Resistance	
**Gumagamit ako ng antibiotics upang mapabilis ang paggaling ko mula sa mga impeksyon.** / I use antibiotics to speed up recovery from infections.	☐ **Oo** / Yes ☐ **Hindi** / No
**Bumibili ako ng antibiotics kahit walang reseta mula sa doktor.** / I buy antibiotics even without a prescription from the doctor.	☐ **Oo** / Yes ☐ **Hindi** / No
**Gumagamit ako ng naitabing antibiotics mula sa mga kaibigan o kamag-anak.** / I used leftover antibiotics from a friend or family member.	☐ **Oo** / Yes ☐ **Hindi** / No<br>**Anong antibiotic ito? Para sa anong karamdaman? Bakit mo ito ginamit?**/ Which antibiotic was this? For what illness? Why did you use them?
**Bumibili ako ng mga may tatak na mamahaling antibiotics.** / I buy the higher priced brand of antibiotics.	☐ **Oo** / Yes ☐ **Hindi** / No<br>**Bakit?** / Why?
**Mahigpit kong sinusunod ang dosis ng antibiotic na inireseta at panahon ng pag-inom ayon sa mga doktor.** / I strictly follow the prescribed dosage and schedule of antibiotics as prescribed by the doctor.	☐ **Oo** / Yes ☐ **Hindi** / No<br>**Kung hindi, paano ka hindi sumusunod sa inireseta? Bakit?**/ If not, how did you deviate from the prescription? Why?

**Naranasan ko nang hindi uminom ng antibiotics sa itinakdang oras ng pag-inom ng dosis.** / I have experienced not taking antibiotics at the prescribed time of dosage.	☐ **Oo** / Yes ☐ **Hindi** / No<br>**Bakit hindi mo sinunod ang itinakdang oras ng pag-inom ng dosis?** / Why did you skip doses?
**Tumitigil ako sa pag-inom ng antibiotics kapag bumubuti na ang aking pakiramdam o nawawala na ang mga sintomas.** / I stop taking antibiotics once I feel better or the symptoms have improved.	☐ **Oo** / Yes ☐ **Hindi** / No<br>**Bakit?** / Why?
**Naranasan ko nang hindi talaban ng antibiotics.** / I have experienced resistance to antibiotics.	☐ **Oo** / Yes ☐ **Hindi** / No<br>**Kung oo, maaari mo bang sabihin ang iyong naging karanasan?** / If yes, can you tell me about your experience?
**Narinig o nabasa ko na ang tungkol sa Antibiotic Resistance o paghina ng talab ng antibiotics.** / I have heard or read about Antibiotic Resistance.	☐ **Oo** / Yes ☐ **Hindi** / No<br>**Kung oo, saan mo narinig at nabasa ang tungkol sa Antibiotic Resistance o paghina ng talab ng antibiotics?** / If yes, where did you hear or read about Antibiotic Resistance?

MARAMING SALAMAT! / THANK YOU!
