# Supplementary material for: Using the socio-ecological model in understanding antimicrobial resistance and antibiotic usage in the lakeshore communities of Calamba and Pila, Laguna, Philippines
Source: Front Public Health. 2026 May 11;14:1827837. doi: 10.3389/fpubh.2026.1827837 (PMC13199311; doi:10.3389/fpubh.2026.1827837)
Supplement: Supplementary file 2 [file Data_Sheet_2.PDF]

Key Informant Interview for Pharmacists
Guide Questions

**Personal na Impormasyon / Personal Information**	
**Pangalan** / Name:	
**Tinapos na digri** / Degree graduated:	
**Taon ng karanasan bilang parmasyutiko** / Years of experience as pharmacist:	
**Tipo ng parmarsya** / Type of pharmacy:	**komunidad** / community **ospital** / hospital **Yunit ng kalusugan sa Kanayunan /** Rural Health Unit (RHU) **iba pa** / others ________

**Paraan ng pagbibigay ng antibiotics / Dispensing antibiotics practices**
**Ang antbiotics ba ay binibibili sa parmasya?** / Are antibiotics being bought from the pharmacy?<br>**Oo** / Yes **Hindi** / No
**Ano ang mas gustong binibili, generic o branded na antibiotics? Bakit?** / What is preferred to buy, generic or branded antibiotics? Why?<br>**generic** **branded**<br><br>**Dahilan** / Reason: ______________________________
**Anu-anong antibiotics ang karaniwang binibili at para sa anong mga sakit?** / Which antibiotics are commonly bought and for what illnesses?
**Kailangan bang may reseta para sa bawat antibiotics na nabanggit na binibili?** / Do you need a prescription for each of the antibiotics mentioned that are being bought?<br>**Oo** / Yes **Hindi** / No
**May mga pagkakataon bang ibinibigay sa mga kliyente ang mga antibiotic na ito kahit walang reseta? Ano ang mga dahilan para dito? /** Are there instances when these antibiotics are given to clients even without prescription? What are the reasons for this?<br>**Oo** / Yes **Hindi** / No<br><br>**Dahilan** / Reason: ______________________________
**May mga kliyente bang minsan na nagtatanong sa inyo kung alin na antibiotic ang dapat nilang bilhin? Ano ang mga pagkakataon kung kailan sila nagtatanong ng inyong opinyon?** / Do clients sometimes ask for your opinion on which antibiotic to purchase? What are the circumstances when they ask for your opinion?<br><br>**Oo** / Yes **Wala** / None<br><br>**Mga pagkakataon** / Circumstances: ______________________________

Nagkaroon na ba ng pagkakataong may mga kliyente na bumibili ng antibiotics para sa kanilang mga alagang hayop o hayop sa sakahan? Kung oo, ano ang mga dahilan kung bakit ginagamit nila ito? / Has there been a time when clients bought antibiotics for their pets or farm animals? If yes, what are the reasons they use them for?

Oo / Yes **Wala** / None

Kung oo, ano ang mga kadahilanan / If yes, what are the reasons:

____

**Kaalaman at Kamalayan sa Paghina ng bisa ng Antibiotics / Knowledge and Awareness of Antibiotic Resistance:**
**Mayroon ka bang kaalaman tungkol sa Paghina ng bisa ng Antibiotic? /** Are you aware of Antibiotic resistance?<br><br>**Oo** / Yes **Wala** / None
**Paano mo nalaman ang mga ito? Ano ang mga pinagkuhanan mo ng impormasyon ukol dito?** / How did you learn about this? What were your sources of information about it?
**Sa palagay mo, kailangan mo pa ba ng karagdagang impormasyon o pormal na pagsasanay tungkol sa paghina ng bisa ng antibiotics? /** Do you think you need more information or formal training on antibiotic resistance?<br><br>**Oo** / Yes **Hindi** / No
**Ano sa tingin mo ang gampanin mo bilang parmasyutiko sa pamimigay ng mga antibiotic? /** What do you think is your role as a pharmacist in dispensing antibiotics?
