# Supplementary material for: Using the socio-ecological model in understanding antimicrobial resistance and antibiotic usage in the lakeshore communities of Calamba and Pila, Laguna, Philippines
Source: Front Public Health. 2026 May 11;14:1827837. doi: 10.3389/fpubh.2026.1827837 (PMC13199311; doi:10.3389/fpubh.2026.1827837)
Supplement: Supplementary file 3 [file Data_Sheet_3.PDF]

Key Informant Interviews

I. Personal information / Personal na impormasyon

Name / Pangalan	
Position / Katungkulan	
Contact Number /	

II. Questions for the / Mga katanungan para sa:

☐ Barangay / ☐ Municipal Health Workers

1. Do the members of your community come to you to ask for antibiotics? / Lumalapit ba sa inyo ang mga tao para humingi ng antibiotic?
2. What were the reasons given when they ask for antibiotics? / Ano ang mga rason sa paggamit ng antibiotic?
3. What types of antibiotics are available here? Do you require prescription from the doctor before giving out antibiotics? / Ano pong mga antibiotic ang available dito? Mayroon po ba kayong hinihinging reseta bago magbigay ng antibiotic?
4. Do community members finish the course of antibiotic medication? If no, why not? When do they usually stop taking the medicines? / Tinatapos ba ng mga tao sa komunidad ang gamutan ng antibitics? Kung hindi, bakit? Kailan o ano ang mga sitwasyon na hindi na nila pinagpapatuloy ito?
5. Are there reported side effects or overuse of antibiotics in your community? Can you elaborate on this? / May mga naitala ba kayo mula sa inyong komunidad na side effect o labis na pag-inom ng antibiotics? Maaari bang higit mo itong ipaliwanag?
6. Is there a program in your barangay or municipality in relation to dissemination of correct information on the use of antibiotics? / Mayroon po bang programa ang barangay / munisipyo kaugnay ng pagpapalaganap ng impormasyon tungkol sa pag-inom ng antibiotic?
7. What do you think would be the most effective way to inform the public about the right information regarding the intake and effects of antibiotics? (Eg. pamphlets, social media posts, conversation with a health worker…) / Ano sa palagay ninyo ang mabisang paraan upang maipaabot sa mga tao ang tamang impormasyon tungkol sa pag-inom ng antibiotic? (Hal. Gamit ang mga babasahin o post sa social media o pakikipag-usap sa mga barangay health worker…)
